# Perpetuating the cycle of violence in South African low-income communities: attraction to violence in young men exposed to continuous threat

**DOI:** 10.3402/ejpt.v7.29099

**Published:** 2016-01-07

**Authors:** Martina Hinsberger, Jessica Sommer, Debra Kaminer, Leon Holtzhausen, Roland Weierstall, Soraya Seedat, Solomon Madikane, Thomas Elbert

**Affiliations:** 1 Department of Psychology, University of Konstanz, Reichenau, Konstanz, Germany; 2 Department of Psychology, University of Cape Town, Cape Town, South Africa; 3 Department of Social Development, University of Cape Town, Cape Town, South Africa; 4 Clinical Psychology and Psychotherapy, Medical School Hamburg, Hamburg, Germany; 5 Department of Psychiatry, Stellenbosch University, Stellenbosch, South Africa; 6 REALISTIC, Cape Town, South Africa

**Keywords:** Victimization, continuous stress, trauma exposure, PTSD, appetitive aggression, attraction to cruelty, violence perpetration, delinquency

## Abstract

**Background:**

Life in the low-income urban communities of South Africa is imprinted by a cycle of violence in which young males predominantly are in the roles of both victim and perpetrator. There is some evidence that adolescents who show an attraction to cruelty can display high levels of psychosocial functioning despite the presence of posttraumatic stress symptoms. However, the role of appetitive aggression in the context of ongoing threats and daily hassles is not yet fully understood.

**Objective:**

In this study, we examine the role of attraction to violence in areas of continuous traumatic stress exposure and its effect on posttraumatic stress disorder (PTSD) severity and violence perpetration.

**Method:**

A sample of 290 young males from two low-income Cape Town communities was surveyed. We assessed appetitive aggression with the Appetitive Aggression Scale (AAS), PTSD symptoms with the PTSD Symptom Scale-Interview, the number of witnessed and self-experienced traumatic event types with an adaptation of the Child Exposure to Community Violence questionnaire, and the number of perpetrated violence event types with an adapted offence checklist from the AAS.

**Results:**

Appetitive aggression scores were predicted by witnessed as well as self-experienced traumatic events. Higher appetitive aggression scores resulted in higher levels of PTSD severity and perpetrated violence.

**Conclusions:**

Young males living in the low-income areas of South Africa may develop an attraction to cruelty in response to exposure to violence. Their willingness to fight in turn can increase the likelihood of continued violent behaviour. In contrast to previous research from postconflict areas, appetitive aggression and engagement in violence do not prevent the development of PTSD, but are instead associated with higher levels of posttraumatic stress. PTSD symptoms such as avoidance and hyperarousal, as well as an attraction to cruelty and thus the willingness to fight, might support survival in areas of ongoing conflict, but at the same time they could fuel the cycle of violence.

I had my first fight when I was 7. I was being beaten up at school, so I escaped home to my parents to tell them what happened. They reacted angrily to my crying. What they did then was handing a knife to me and sending me back to the boys to fight them. So I did … (Former perpetrator and drug addict, now a student at the Realistic Life & Skill Training Centre) It is very easy to become a gang member and nearly impossible to leave again. There are only two ways of escaping the cycle of violence after joining a gang: dying or trying to be forgotten. (Former gang member, now a co-worker at the Realistic Life & Skill Training Centre)

Life in the low-income urban communities of South Africa is characterized by a cycle of violence. This cycle is evident in the presence of rival gangs that are locked in a never-ending pattern of attack and counterattack (Dixon & Johns, 2001) and in the community that attempts to reduce crime by acts of vigilantism, also called “mob justice” (Buur & Jensen, 2004). Executions in the form of stoning, burning, mutilation, and similar retaliatory acts can arise spontaneously after an alleged criminal has been hunted down and caught by community members. However, there are also more organized forms of vigilantism through community-based crime-fighting institutions like PAGAD (People Against Gangsterism and Drugs), which first started as a popular movement, developed into a vigilante group, and gradually became an urban terror organization (Dixon & Johns, 2001). Crime and the violent methods of crime reduction have resulted in an environment of permanent threat for all inhabitants in the community.

Young men are not only perpetrators in this scenario but also victims (Kaminer, Du Plessis, Hardy, & Benjamin, 2013; Norman, Matzopoulos, Groenewald, & Bradshaw, 2007; Seedat, Van Niekerk, Jewkes, Suffla, & Ratele, 2009). According to Norman et al. (2007), homicide is the leading cause of fatal injury in South African men, at a rate seven times higher than that for women. The numbers are highest for those in the age group of 15–29 years, with homicide rates of 184 per 100,000, which equates to nine times the global rate. Kaminer, Hardy, Heath, Mosdell, and Bawa (2013) questioned 230 Xhosa-speaking adolescents from an urban public high school about their traumatic experiences and found that boys had higher scores than girls for trauma exposure in the community and at home and for witnessing domestic violence in particular. Boys even reported significantly higher rates of sexual abuse in their families than girls. In addition, the frequency and severity of beatings that children are exposed to at home were greater for boys than for girls (Seedat et al., 2009). Boys are also at a higher risk of polyvictimization (Kaminer, du Plessis, et al., 2013).

Living in a community where violence is prevalent can cause anxiety disorders such as posttraumatic stress disorder (PTSD), aggression, and externalizing behaviour (Elbert, Rockstroh, Kolassa, Schauer, & Neuner, 2006; Fowler, Tompsett, Braciszewski, Jacques-Tiura, & Baltes, 2009; Van der Merwe & Dawes, 2000) and thus give rise to both revictimization and the intergenerational cycling of violence (Seedat et al., 2009). Single traumas usually do not lead to the development of PTSD (Breslau, 1998), but continuous exposure to traumatic stressors is associated with an increased risk of PTSD in adults (Neuner et al., 2004) as well as children (Catani et al., 2009; Catani, Jacob, Schauer, Kohila, & Neuner, 2008). PTSD rates in adolescents from the low-income areas of South Africa have been found to be between 20 and 25% (Seedat, Nyamai, Njenga, Vythilingum, & Stein, 2004; Suliman et al., 2009), which is at least three times the rates of European and North American adolescents (Kessler, Sonnega, Bromet, Hughes, & Nelson, 2005; Perkonigg, Kessler, Storz, & Wittchen, 2000). Sexually abused boys are at higher risk of later becoming sexual abusers themselves (Aebi et al., 2015), and those who have witnessed violence against their mothers are more likely to become perpetrators of intimate partner violence (Hotaling & Sugarman, 1986) and community violence (Jewkes & Abrahams, 2002; Shields, Nadasen, & Pierce, 2009). The transformation from victim to perpetrator is an adaptation that entails advantages for those who have begun to feel attracted to cruelty. Appetitive aggression is described as the violence-related enjoyment a perpetrator experiences through his or her acts of violence or inflicting harm on a victim (Elbert, Weierstall, & Schauer, 2010). In contrast to “reactive aggression,” which has an affective, defensive, and retaliatory nature, appetitive aggression falls into the category of “instrumental aggression,” which is said to be more proactive, predatory, and goal-directed. Appetitive aggressive behaviour (including extreme forms of aggression and violence taking place in conflict and war) is characterized by the fuelling of violence and a fascination with—sometimes even enjoyment of—cruelty. Several studies in African high- and postconflict settings have found that participants with higher levels of appetitive aggression had a higher military rank (Crombach, Weierstall, Hecker, Schalinski, & Elbert, 2013; Hermenau, Hecker, Maedl, Schauer, & Elbert, 2013), felt a greater closeness to their comrades (Haer, Banholzer, Elbert, & Weierstall, 2013), and were preferred by women as short-term mates, especially during the fertile window of the menstrual cycle (Giebel, Weierstall, Schauer, & Elbert, 2013). Additionally, attraction to cruelty has been identified as a factor that protects against the development of PTSD after combat (Weierstall, Huth, Knecht, Nandi, & Elbert, 2012; Weierstall, Schaal, Schalinski, Dusingizemungu, & Elbert, 2011; Weierstall, Schalinski, Crombach, Hecker, & Elbert, 2012). The possible advantages of an attraction to violence in low-income urban South African communities were investigated by Weierstall, Hinsberger, et al. (2013) in a sample of 69 male ex-offenders; their research revealed that participants scoring high with regard to appetitive aggression exhibited better functioning and expressed fewer concerns about future threats in comparison to adolescents who only exhibited reactive aggression.

Children who are exposed to and forced to engage in violent behaviour in wartime often apply violent behaviour to resolve their conflicts even after relocation to their home villages after the war has ended (Schauer & Elbert, 2010). Studies on Burundian street children (Crombach & Elbert, 2014) and young refugees from various countries (Mueller-Bamouh, Ruf-Leuschner, Dohrmann, Schauer, & Elbert, in press) have shown that children with high levels of appetitive aggression were more likely to display aggressive behaviour than those with low levels. Conversely, violent behaviour predicted higher levels of the enjoyment of cruelty (Crombach et al., 2013; Hecker, Hermenau, Mädl, Elbert, & Schauer, 2012; Weierstall et al., 2011; Weierstall, Haer, Banholzer, & Elbert, 2013; Weierstall, Schalinski, et al., 2012). Hence, the cycle of violence also seems to manifest itself in the way that attraction to violence results in more self-committed violent acts, which again lead to greater enjoyment of cruel behaviour, and so forth. Consequently, the re-integration of former child soldiers, ex-combatants, violent street children, and ex-offenders into society can be difficult, and their acceptance by their communities low (Schauer & Elbert, 2010; Sommer et al., in press). An adaptation involving the development of an attraction to violent behaviour thus entails not only advantages but also disadvantages for the perpetrators; in consideration of the level of vigilantism in South Africa, these disadvantages may even be deadly.

## Objective

The aim of the present study was to examine the role of attraction to violence in areas of continuous traumatic stress exposure. Firstly, we postulate that higher levels of (witnessed as well as self-experienced) continuous traumatic stress will predict higher levels of appetitive aggression, as found in a study on Ugandan ex-child soldiers in a postconflict area (Weierstall, Schalinski, et al., 2012). Secondly, we seek to confirm the finding of Weierstall, Hinsberger, et al. (2013) with a larger South African sample that an attraction to cruelty does not protect individuals from the development of posttraumatic stress as it does in postconflict areas, but instead increases it in areas of continuous stress. Thirdly, we additionally postulate that in postconflict areas (Crombach, & Elbert, 2014; Mueller-Bamouh et al., in press), a stronger attraction to cruelty will lead to more offences in areas of ongoing threat.

## Method

### Participants

The 290 participants in the study, all originally from the suburbs of Gugulethu and Khayelitsha, Cape Town, South Africa, were exclusively male and native Xhosa speakers. The age range was from 14 to 40 years, with a mean of 22 years (*SD*=4.5). A total of 80.7% had no matriculation, 17.9% had a matriculation, and 1.4% had a college degree. The mean number of years of education was 10.5 years (*SD*=1.77). All participants were recruited with the help of staff at the Rebuilding and Life Skill Training Centre (REALISTIC) located in Gugulethu and Khayelitsha. The goal of this programme is the re-integration of former juvenile offenders into society, family, and work life.

Fifty-one percent of the sample were current or former participants in a re-integration programme, and 49% have never participated in any re-integration programme. Participation in the programme was either voluntary (i.e., adolescents with a desire to change their lives in terms of drug abuse and violent behaviour) or obligatory (i.e., adolescents sent by worried family members or referred by the police station in lieu of other punitive measures).

All participants gave informed and written consent. In the case of under-aged participants, parents or caretakers were additionally asked to give their written consent. The study protocol including these consent forms was approved by the Ethical Review Boards of Stellenbosch University, South Africa, the University of Cape Town, South Africa, and the University of Konstanz, Germany.

The participants’ sociodemographic data are summarized in Table 1.

**Table 1:** Sociodemographic data of the 290 South African study participants

Sociodemographic data	(*n*=290)
Age, median (*SD*) [range]	21 (4.5) [14–40]
Years of formal education, mean (*SD*) [range]	10 (1.8) [1–16]
Highest completed education, no. (%)	
None	7 (2.4%)
Primary school	227 (78.3%)
Secondary school	52 (17.9%)
College	4 (1.4%)
Participation in a re-integration programme	
Yes (formerly or current), no. (%)	148 (51%)
Never, no. (%)	142 (49%)

### Interviews

The interviews were mainly conducted in an office building in Salt River, Cape Town. Transport to Salt River was organized for all participants from the REALISTIC offices in Gugulethu. Interviews took place from the end of October 2013 until mid-December 2013 and from the beginning of February 2014 to the beginning of March 2014. Interviews took 2 h on average, and participants were reimbursed with ZAR100 (roughly US$8.50).

Diagnostic interviews were carried out by a group of four German mental-health experts and three local counsellors, who had received a 25-h training of an expert psychologist in the theoretical concepts of mental disorders, trauma and aggression, and clinical diagnosis. Interviewees were encouraged to speak in either English or isiXhosa. A trained interpreter accompanied English-speaking interviewers. These interpreters were native isiXhosa speakers who were fluent in English.

Back-and-forth translations of the questionnaires were used to generate bilingual surveys, starting with a translation from English to isiXhosa, followed by back-translation into English by a different interpreter. These translations were discussed with the interpreters in a multiprofessional team (including two native isiXhosa-speaking South Africans from the Cape Town suburb of Gugulethu who had been working in the community for decades) until there was consensus on each item. A clinical psychologist trained the interpreters in the concepts of trauma, posttraumatic stress, and proactive, reactive, and appetitive aggression in an intensive training course in order to improve the accuracy and validity of the translations. Regular individual and team supervision ensured cross-interview consistency and psychohygiene.

### Measures

#### Trauma exposure

To measure the amount of exposure to traumatic stressors, a 36-item list adapted from the Child Exposure to Community Violence (CECV) instrument (Amaya-Jackson, 1998) was used. The CECV event list is a 33-item self-reported checklist that assesses children's levels of witnessing, hearing about, or experiencing violence. The checklist was adapted from Richters and Martinez's (1993) “Things I've seen and heard” and was designed to reflect the types of violence to which adolescents in low-income South African areas are commonly exposed (e.g., robbery, assault, stabbings, shootings, and sexual abuse). The CECV is reported to show good internal consistency and test–retest reliability (Fehon, Grilo, & Lipschitz, 2001). It has also been used in previous research on South African youth (Fincham, Altes, Stein, & Seedat, 2009; Weierstall, Hinsberger, et al., 2013).

For each trauma event type, participants were asked whether the incident had happened to them or in their presence as a child and/or an adult. “Child” in this questionnaire was defined as aged 0–15, and “adult” was defined as aged 16 and above (i.e., the age at which full membership in a gang would be possible). Events could either be self-experienced or witnessed and were scored with 1=“experienced/witnessed” or 0=“not experienced/witnessed.” The sum of the experienced and witnessed event types represents the severity of an individual's exposure to traumatic events and community violence (*witnessed/self-experienced trauma event types*). Sum scores for witnessed violence ranged from 2 to 16 (out of 16 different types of stressful or traumatic events). The average number of witnessed traumatic event types was 10.2 (*SD*=2.6); the median was 10. Nineteen items on the trauma event list were summed up to measure the number of *self-experienced stress event types*. Participants experienced at least 1 and up to 16 different types of traumatic events. On average, they were exposed to 8.4 (*SD*=3.0) traumatic event types, with a median of nine event types. The frequency distribution of the respective items is found in Table 2.

**Table 2:** Frequencies of the different types of witnessed and self-experienced trauma events (n=290)

List of traumatic event types	
*Witnessed violence event types*	*Prevalence (%)*
Have you ever witnessed someone being physically attacked by someone else?	98.6
Have you ever witnessed someone being attacked with a weapon by someone else?	95.2
Have you ever witnessed someone being threatened (to be harmed) by someone else?	91.7
Have you ever seen a dead body (besides at funerals)?	90.7
Was someone you know killed by another person?	81.4
Did someone close to you suffer from a serious illness?	73.7
Have you ever seen somebody being killed?	72.8
Have you ever witnessed a bad accident, like a very serious car accident?	67.8
Have you ever witnessed someone being tortured?	65.3
Have you ever witnessed a life-threatening fire or explosion?	63.8
Have you ever witnessed a family member being attacked by another family member?	59.0
Have you ever witnessed a painful and scary medical treatment (e.g., during an initiation)?	51.0
Have you ever witnessed a family member being threatened by another family member?	42.9
Have you ever witnessed a family member being attacked with a weapon by another family member?	36.8
Have you ever witnessed someone being sexually assaulted by someone else?	26.0
Have you ever witnessed sexual assault in your family by another family member?	2.4
*Self-experienced violence event types*	*Prevalence (%)*
Have you ever been threatened to be harmed by someone outside your family?	88.3
Have you ever been physically attacked by someone else?	86.2
Have you ever been attacked with a weapon by someone else?	84.5
Have you ever been physically attacked by someone in your family?	80.0
Have you ever lost a parent/caregiver?	58.5
Have your parents/caregivers regularly humiliated you verbally (e.g., insulted you; said you're worthless or a bad child)?	55.0
Have you ever been attacked with a weapon (e.g., stick, stone, bottle, belt, knife, gun) by a family member?	51.7
Have you ever felt neglected by your parents/caregivers (e.g., they didn't support you; didn't send you to school even though they could have; didn't care for you)?	49.1
Have you ever had a painful and scary medical treatment (e.g., during an initiation or in a hospital, when you were sick or badly injured)?	47.4
Have you ever been imprisoned?	41.9
Have you ever severely suffered from hunger, so that you worried about your health?	39.9
Have you ever been threatened to be harmed by someone in your family?	37.7
Have you ever been tortured?	37.2
Have you ever suffered from a serious illness?	27.4
Have you ever been in a bad accident, like a very serious car accident?	22.6
Have you ever been in a life-threatening fire or explosion?	14.2
Have you ever been in any kind of natural disaster (e.g., a fire, a tornado/hurricane, a flood, an earthquake)?	9.7
Have you ever been sexually assaulted by someone else?	5.6
Have you ever been sexually assaulted by a family member (e.g., abuse, doing something with your or their private parts that you didn't want to, watching porn although you were too young or didn't want to)?	2.1

#### Posttraumatic stress symptom severity

The severity of PTSD symptoms was assessed with the PTSD Symptom Scale-Interview (PSS-I; Foa & Tolin, 2000), which covers the 17 PTSD symptoms according to the DSM-IV (American Psychiatric Association [APA], 2000) and asks respondents about their symptom intensity during the past 2 weeks. It has also been used in African samples (e.g., Ertl et al., 2011; Hecker et al., 2012; Jacob, Neuner, Maedl, Schaal, & Elbert, 2014; Köbach, Schaal, & Elbert, 2015). For the assessment of PTSD symptoms, participants were asked to identify the most traumatic event that had happened in their lives that was still bothersome. Both the subjective and the objective A-criteria were used in estimating the PTSD rates. All symptoms were rated from 0 (= “not at all/only once”) to 3 (= “five or more times per week/almost always”). For computation of the *severity of PTSD*, the frequencies of all 17 PTSD symptoms were summed. The participants’ PTSD sum scores ranged from 0 to 39 out of a maximum possible score of 51 points. The mean score was 8.5 points (*SD*=9.1), and the median was 5 points. A total of 19.5% of the 257 fully diagnosed participants fulfilled the DSM criteria for PTSD. Thirty-three PTSD ratings were not available due to rater errors. The PSS-I manifested excellent internal consistency values (Cronbach's *α=*0.88) and high inter-rater reliability (intraclass correlation coefficient, ICC=0.86) in this study.

#### Attraction to violence

Attraction to violence was measured by the Appetitive Aggression Scale (AAS; Weierstall & Elbert, 2011), which has demonstrated good psychometric properties in various violent populations. The Cronbach's *α* coefficient for the South African sample for this measure was 0.86, and the inter-rater reliability was ICC=0.84. The questionnaire consists of 15 questions on instrumental aggression (“Do you enjoy inciting your fellows to fight?”), addiction-specific questions (“Once fighting has started, do you get carried away by the violence?”) that cover the reward-driven aspect of appetitive aggression, and questions about the desire to cause harm (“Once you got used to being cruel, did you want to be crueller and crueller?”). Responses are rated on a 5-point Likert scale (0=“disagree totally” to 4=“agree totally”). The AAS score is then calculated by summing the scores of the 15 items; possible scores range from 0 to 60. The mean appetitive aggression score in the sample was 15.4 points (*SD*=13.1), and the median was 12 points.

#### Perpetrated violence

The score for perpetrated violence was calculated from the number of 21 different offence event types. The list of these self-committed violence types was adapted from the AAS and has previously been successfully administered in a population of South African juvenile offenders (Weierstall, Hinsberger, et al., 2013). The items reflect a range of violence, starting with event types of little impact (“Have you shouted at someone?”; “Have you slapped someone?”) and progressing to severe criminal acts (“Have you mutilated someone?”; “Have you raped someone?”). Possible sum scores ranged from 0 to 21. The average score was 11.7 points (*SD*=4.4), and the median score was 12 points, with a range of 1–21. In this current study's sample, the Cronbach's *α* coefficient was 0.92, and the inter-rater reliability was ICC=0.85.

### Data analysis

Spearman correlations between witnessed and self-experienced trauma event types, PTSD symptom severity, appetitive aggression, and perpetrated violence types were calculated using SPSS 21. To investigate the complex interactions between predictor and outcome variables, further path analysis was conducted using AMOS 22. Witnessed violence event types and self-experienced violence event types were assessed as predictor variables. PTSD symptom severity and perpetrated violence event types were processed as outcome variables. Attraction to violence was the outcome variable for witnessed and self-experienced stress and the predictor variable for PTSD severity and perpetrated violence. The level of significance was set to *α*=5%.

## Results

### Correlations between different trauma event types, PTSD severity, appetitive aggression, and perpetrated violence types

A correlation matrix of all five variables (self-experienced and witnessed trauma event types, PTSD symptom severity, attraction to violence, and perpetrated violence types) reveals significant Spearman correlations (according to Cohen, 1988) between all of the variables. The correlation coefficients, levels of significance, and population size are presented in Table 3.

**Table 3:** Correlation matrix of witnessed trauma event types, self-experienced trauma event types, PTSD severity, attraction to violence, and perpetrated violence types

	Sum of witnessed trauma event types	Sum of self-experienced trauma event types	Sum of PTSD symptom scores	Sum of appetitive aggression score
Sum of self-experienced trauma event types	0.61*** (*p*<0.001)	—		
Sum of PTSD symptom scores	0.29*** (*p*<0.001)	0.36*** (*p*<0.001)	—	
Sum of appetitive aggression score	0.35*** (*p*<0.001)	0.40*** (*p*<0.001)	0.28*** (*p*<0.001)	—
Sum of perpetrated violence types	0.36*** (*p*<0.001)	0.38*** (*p*<0.001)	0.29*** (*p*<0.001)	0.53*** (*p*<0.001)

*** *p*<0.001; correlation is significant at the 0.01 level (two-tailed).

### The development and outcomes of appetitive aggression in a context of ongoing threat

To further investigate the role of attraction to violence in a context of continuous stress, we conducted a path analysis with witnessed and self-experienced traumatic events as outcome variables representing the ongoing threat. Attraction to violence, actual violent behaviour, and PTSD symptom severity were chosen as outcome variables.

Attraction to violence was predicted by witnessed traumatic incidents as well as victimization. Self-committed offences, however, were directly predicted only by the witnessing of violence. In contrast, PTSD symptom severity was directly predicted by victimization only. The witnessing and self-experience of violence had an indirect influence on both PTSD severity and perpetrated violence, via appetitive aggression. Appetitive aggression itself predicted both the severity of posttraumatic stress and aggressive behaviour. The graph below displays all of the results.

Beta coefficients are statistically significant at *p*<0.05 for the effect of witnessed trauma event types on attraction to violence as well as the effect of attraction of violence on PTSD symptom severity. All other beta coefficients were significant at a level of *p*<0.001. According to the criteria for an adequate model fit (Browne & Cudeck, 1993; Carmines & McIver, 1981; Wheaton, Muthén, Alwin, & Summers, 1977), the path model displayed below fulfils the requirements, *χ*^2^(3)=6.681, *p=*0.083, *χ*^2^/df=2.23, comparative fit index=0.989, root-mean-square error of approximation=0.065.

## Discussion

The association between an environment of ongoing threat and the development of appetitive aggression has been investigated in former Ugandan child soldiers in a study by Weierstall et al. (2011), with the researchers determining that witnessing violence predicted higher levels of attraction to violence. The influence of victimization was not examined. Our results confirm the connection between witnessing violence and attraction to it, but also show that the self-experience of violence is an even stronger predictor for appetitive aggression. According to our results, appetitive aggression in turn predicts the level of self-committed violence. Those findings are in line with Athens’ theory on the process of violentization (Athens & Ulmer, 2003), which explains that self-experienced violent acts (“violent subjugation”) in combination with the isochronic observation of violent acts (“horrification”) results in what Athens calls “brutalization.” Later stages of Athens’ model describe how a child gradually begins to act more and more violently—at first, only when provoked (“defiance”), but if the use of violence is successful, it becomes a preferred method of self-protection (“violent performance”) and ultimately a preferred instrument for the resolution of all kinds of problems (“virulency”). In a context of ongoing threat, not hesitating to use violence and the failure to experience subsequent feelings of anxiety or guilt (but instead actually enjoying it) can be an evolutionary advantage, ensuring survival and psychosocial functioning.

As seen in other contexts of high exposure to violence, attraction to cruelty can even prevent a combatant from developing posttraumatic stress symptoms after conflict has ceased (Weierstall et al., 2011; Weierstall, Huth, et al., 2012; Weierstall, Schalinski, et al., 2012). However, in the South African context of continuous threat, attraction to cruelty is associated with higher levels of posttraumatic stress. PTSD symptoms seem to remain at a consistent level over the years when strong feelings of revenge are present (Gäbler & Maercker, 2011; Orth, Montada, & Maercker, 2006), whereas the symptoms decrease when these feelings are not present (Orth et al., 2006). Young men in the low-income areas of South African cities, especially those who are members of criminal street gangs, tend to obey rules like “blood in–blood out,” which requires them to avenge the murder of a friend or fellow gang member by killing the perpetrator or someone else from the rival gang (Commonwealth of Virginia Department of State Police Virginia Fusion Center, 2008). The resulting desire for revenge and the preoccupation with thoughts of retaliation could trigger memories of traumatic incidents and (in the same way) also prolong other symptoms of posttraumatic stress. Additionally, high levels of posttraumatic symptoms such as hyperarousal or avoidance could have an important protective function in a context of continuous threat, enhancing an individual's chances of survival. These results support the findings of Weierstall, Hinsberger, et al. (2013), who demonstrated that former South African offenders with high levels of appetitive aggression and high levels of PTSD symptoms had higher levels of psychosocial functioning than those who were not enjoying violence perpetration but only acted aggressive in a reactive fashion.

This study relied on self-reports. Sexual victimization is likely to be under-reported (Jewkes & Abrahams, 2002; Kaminer, du Plessis, et al., 2013 2013). Sieverding (2002) has suggested that males are less likely to report physical and psychiatric symptoms due to the stereotypical “machismo” male gender role that views the reporting of physiological and/or psychological symptoms as “unmanly.” In addition, the commission of certain aggressive acts, such as rape or the desecration of dead bodies, might be under-reported due to social undesirability. However, the subjective underestimation of aggression would not have affected correlations or the path model, which suggests that our findings are likely to be valid.

**Fig. 1:**
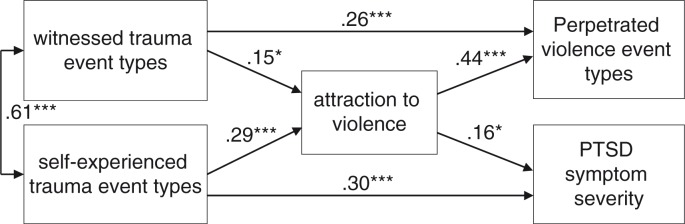
Path model presenting the results of an AMOS path analysis, showing standardized regression weights and significance levels for the relationships between witnessed and self-experienced trauma event types, attraction to violence, PTSD symptom severity, and perpetrated violence types. *p<0.05, **p<0.001.

Finally, the AMOS path model allows for the assessment of correlations and predictors but does not necessarily provide evidence of causal relationships between the observed variables (Fig. 1).

## Conclusions

We have conclusively determined that high levels of appetitive aggression and thus a preparedness and willingness to resort to violence, as well as PTSD symptoms such as hyperarousal and avoidance, can lead to better chances of survival in a context of continuous threat and thus could fulfil an important role in survival and functioning in such an environment. At the same time, the willingness to fight can lead to suffering in the community as well as in the perpetrator. Young offenders are at the highest risk of getting killed or being further traumatized by rival gangs, the police, or vigilantes in the community. They become excluded from society, and thereby their chances of leading a normal social life (graduating from high school, finding a job, etc.) further decrease. In addition, the agonizing symptoms of posttraumatic stress (including nightmares, flashbacks, and sleep and concentration disturbances) are possibly maintained at a high level and might serve as an additional stressor. The aggressive behaviour of these young offenders can fuel the cycle of violence in the communities. Its protective nature notwithstanding, appetitive aggression may breed more violence, and thus interventions aimed at the reduction of violence would seek to reduce the attraction to violence. At the same time, it is important to provide alternative skills to young men growing up under conditions of continuous threat that will allow them to develop more productive and less destructive problem-solving methods.
